# Association of glutamate receptor gene polymorphisms with attention-deficit hyperactivity disorder susceptibility: a systematic review and meta-analysis

**DOI:** 10.3389/fgene.2024.1348387

**Published:** 2024-03-13

**Authors:** Dehua Zou, Qiaoli Zeng, Pei Liu, Yue Wei, Runmin Guo, Yizhun Zhu, Rong-Rong He

**Affiliations:** ^1^ School of Pharmacy, Macau University of Science and Technology, Macau, Macau SAR, China; ^2^ Guangdong Engineering Research Center of Chinese Medicine and Disease Susceptibility, Jinan University, Guangzhou, Guangdong, China; ^3^ Department of Internal Medicine, Shunde Women and Children's Hospital (Maternity and Child Healthcare Hospital of Shunde Foshan), Guangdong Medical University, Foshan, Guangdong, China; ^4^ Key Laboratory of Research in Maternal and Child Medicine and Birth Defects, Guangdong Medical University, Foshan, Guangdong, China; ^5^ Matenal and Child Research Institute, Shunde Women and Children’s Hospital (Maternity and Child Healthcare Hospital of Shunde Foshan), Guangdong Medical University, Foshan, Guangdong, China; ^6^ State Key Laboratory of Quality Research in Chinese Medicine, Macau University of Science and Technology, Macau, Macau SAR, China; ^7^ Department of Ultrasound, Shunde Women and Children’s Hospital (Maternity and Child Healthcare Hospital of Shunde Foshan), Guangdong Medical University, Foshan, Guangdong, China

**Keywords:** attention-deficit hyperactivity disorder, glutamate receptor gene, rs2284411, rs2229193, rs3792452, meta-analysis

## Abstract

**Background:** There is a growing body of evidence indicating a possible association between genetic variations and attention-deficit hyperactivity disorder (ADHD), although the results have been inconsistent. The objective of this study was to evaluate the correlation between the GRIN2A, GRIN2B and GRM7 gene polymorphisms and ADHD.

**Methods:** A comprehensive meta-analysis and subgroup evaluation was conducted using a fixed-effects model to analyze the association between ADHD and GRIN2B (rs2284411), GRIN2A (rs2229193), and GRM7 (rs3792452) in six genetic models (dominant, recessive, overdominant, homozygous, heterozygous, and allele models).

**Results:** The meta-analysis comprised 8 studies. The overall analysis showed that the GRIN2B rs2284411 T allele and T carries were significantly associated with a decreased risk of ADHD (dominant model:TT + CT vs. CC: OR = 0.783; 95% CI: 0.627–0.980; *p* = 0.032, allele model:T vs. C: OR = 0.795; 95% CI: 0.656–0.964; *p* = 0.019), especially in the Korean subgroup (dominant model:TT + CT vs. CC: OR = 0.640; 95% CI: 0.442–0.928; *p* = 0.019, overdominant model: CT vs. TT + CC: OR = 0.641; 95% CI: 0.438–0.938; *p* = 0.022, allele model:T vs. C: OR = 0.712; 95% CI: 0.521–0.974; *p* = 0.034 and heterozygous model: CT vs. CC: OR = 0.630; 95% CI: 0.429–0.925; *p* = 0.018). However, no meaningful associations were found for rs2229193 and rs3792452.

**Conclusion:** The results of the meta-analysis provide strong evidence that the rs2284411 T allele is significantly associated with reduced susceptibility to ADHD, particularly in the Korean population.

## 1 Introduction

Attention-deficit hyperactivity disorder (ADHD) is widespread childhood neurodevelopmental disorder characterized by hyperactivity, impulsivity, and inattention. The prevalence of ADHD in children is 2.6%–4.5% worldwide (Polanczyk et al., 2015), and it is generally thought to be the result of genetic and environmental interactions (Thapar and Cooper, 2016). Previous studies have indicated that genetic factors play an important role in the pathogenesis of ADHD, with an estimated heritability ranging from 60% to 90% (Gallo and Posner, 2016). Although the precise etiology of ADHD remains uncertain. One study found that dysregulation of neurotransmitter systems is associated with cognitive and motor deficits and the pathogenesis of ADHD (Polanczyk et al., 2007).

Glutamate is the main excitatory neurotransmitter for approximately 60% of neurons in the brain. Glutamate receptors transmit the largest number of excitatory synapses in the central nervous system (Ozawa et al., 1998). Given this important role in neuronal correspondence and synaptogenesis, glutamate receptors play a significant role in cognitive processes (Riedel et al., 2003). Glutamate affects the central nervous system by binding to ionotropic or metabotropic receptors.

Ionotropic glutamate receptors (iGluRs) are classified as NMDA receptors, AMPA receptors, KA receptors, and orphan receptors based on their affinity for glutamate (Hadzic et al., 2017). When glutamate or other stimulants act on iGluRs, the receptors undergo a conformational change, opening channels in the cell membrane and activating the receptors. This activation leads to the influx of Na^+^, K^+^, and/or Ca^2+^ ions, which in turn activate the corresponding signaling pathways. iGluRs are the main excitatory neurotransmitter receptors in the central nervous system. They are involved in synaptic transmission, neuromodulation, and synaptic plasticity, making them important in neurophysiology (Huang et al., 2019).

Molecular genetic research into the development of ADHD has largely centered on association analysis of functional candidate genes. Both genome-wide association studies (GWAS) and candidate gene studies have focused on the dopaminergic pathway, the adrenergic pathway, the serotonin pathway, the cholinergic pathway, and the neurodevelopmental pathway (Hawi et al., 2015). However, GWAS studies have yielded few positive results, and gene enrichment analysis has indicated that signals associated with GWAS are primarily found in neurodevelopmental pathways (Hawi et al., 2015).

Recent studies using GWAS have indicated that the glutamatergic neurotransmitter system, particularly iGluRs, is linked to ADHD, affecting both disease susceptibility and clinical symptoms. The N-methyl-D-aspartate (NMDA) receptor subunits GRIN2A and GRIN2B are glutamate ionotropic receptors that play a role in learning and memory by regulating synaptic plasticity (Sakimura et al., 1995; Tang et al., 1999). Research has shown that genetic variants in the GRIN2A and GRIN2B genes may be involved in ADHD susceptibility (Smalley et al., 2002; Adams et al., 2004; Turic et al., 2004). Additionally, it has been found that GRIN2B SNPs show significant biased transmission, with rs2284411 showing a clear association in families with ADHD (Dorval et al., 2007). Furthermore, individuals with the CC genotype of GRIN2B rs2284411 have been observed to respond better to methylphenidate treatment (Kim et al., 2016).

In addition, the glutamate metabotropic receptor mGluR7 (GRM7) is a type of G-protein-coupled receptor. Interestingly, the GRM7 gene is extensively expressed in the cerebellum, hippocampus, and cerebral cortex, and previous studies have shown an association between ADHD and different structures in these regions (Kinoshita et al., 1998; Kosinski et al., 1999). Moreover, GRM7 may play a role in working memory, fear responses, and anxiety (Masugi et al., 1999; Cryan et al., 2003; Callaerts-Vegh et al., 2006; Fendt et al., 2008). ADHD has been identified as a potential major factor in the development of working memory deficits and anxiety. A study examined the methylphenidate response in childhood ADHD in relation to a SNP (rs3792452) in the GRM7 gene (Mick et al., 2008).

Therefore, GRIN2A, GRIN2B, and GRM7 may be candidate genes for the development of ADHD. However, the results of studies investigating SNPs in glutamate receptor genes are conflicting. Therefore, the aim of this study was to investigate the association of ADHD with three glutamate receptor gene SNPs (GRIN2A rs2229193, GRIN2B rs2284411 and GRM7 rs3792452). We performed a meta-analysis to estimate the association of these three glutamate gene polymorphisms and ADHD.

## 2 Materials and methods

The PRISMA (Preferred Reporting Items for Systematic Reviews and Meta-Analyses) guidelines were followed in this study.

### 2.1 Literature search

Studies were systematically searched in the Chinese National Knowledge Infrastructure, PubMed, and Google Scholar database using the following terms.

1) “GRIN2A” or “rs2229193”or " rs8049651”and “ADHD”;
2) “GRIN2B” or “rs2284411”and “ADHD”; and
3) “GRM7”, or “rs3792452”and “ADHD.”

There were no restrictions on time or language during the search. Studies were assessed by reviewing their titles and abstracts, and irrelevant ones were removed. The complete text of the qualified studies was then evaluated thoroughly to ensure their inclusion.

### 2.2 Inclusion and exclusion criteria

The study’s inclusion criteria required (a) cohort or case-control studies evaluating the link between rs2229193, rs2284411, or rs3792452 and ADHD risk; (b) ample raw or calculated data to determine odds ratios (ORs) and corresponding 95% confidence intervals (CIs); and (c) ADHD diagnoses following the Diagnostic and Statistical Manual of Mental Disorders, fourth edition, DSM-IV (APA, 2000).

The study’s exclusion criteria were: (a) the absence of a case-control design; (b) no connection with rs2229193, rs2284411, or rs3792452 in relation to ADHD risk; (c) inadequate data; and (d) the control group not being in Hardy-Weinberg equilibrium (HWE).

### 2.3 Data extraction

The included studies were subject to independent data extraction to ensure the authenticity of the following information: first author, year, population, number of cases and controls, allele distribution, genotype distribution, HWE, and other relevant data. Studies lacking this information were excluded.

### 2.4 Statistical analysis

Six genetic models were examined for rs2284411, rs2229193 and rs3792452. Genetic heterogeneity was measured using the I^2^-test and the Q-test. Heterogeneity was defined as *p* > 0.1 and I^2^ < 50% using the Mantel–Haenszel fixed effects model to calculate ORs with corresponding 95% CIs. If not, the Mantel–Haenszel random effects model was adopted (COCHRAN, 1950; DerSimonian and Laird, 1986; Higgins et al., 2003). The significance of ORs was evaluated using Z tests. To determine the influence of individual data-sets on the pooled OR, sensitivity analysis was conducted by removing each study from each meta-analysis (Riley et al., 2004). Publication bias was assessed through Begg’s and Egger’s tests (Begg and Mazumdar, 1994; Egger et al., 1997). All statistical analyses were performed using Stata v.16.0 software (Stata Corporation, Texas).

## 3 Results

### 3.1 Study inclusion and characteristics

**FIGURE 1:**
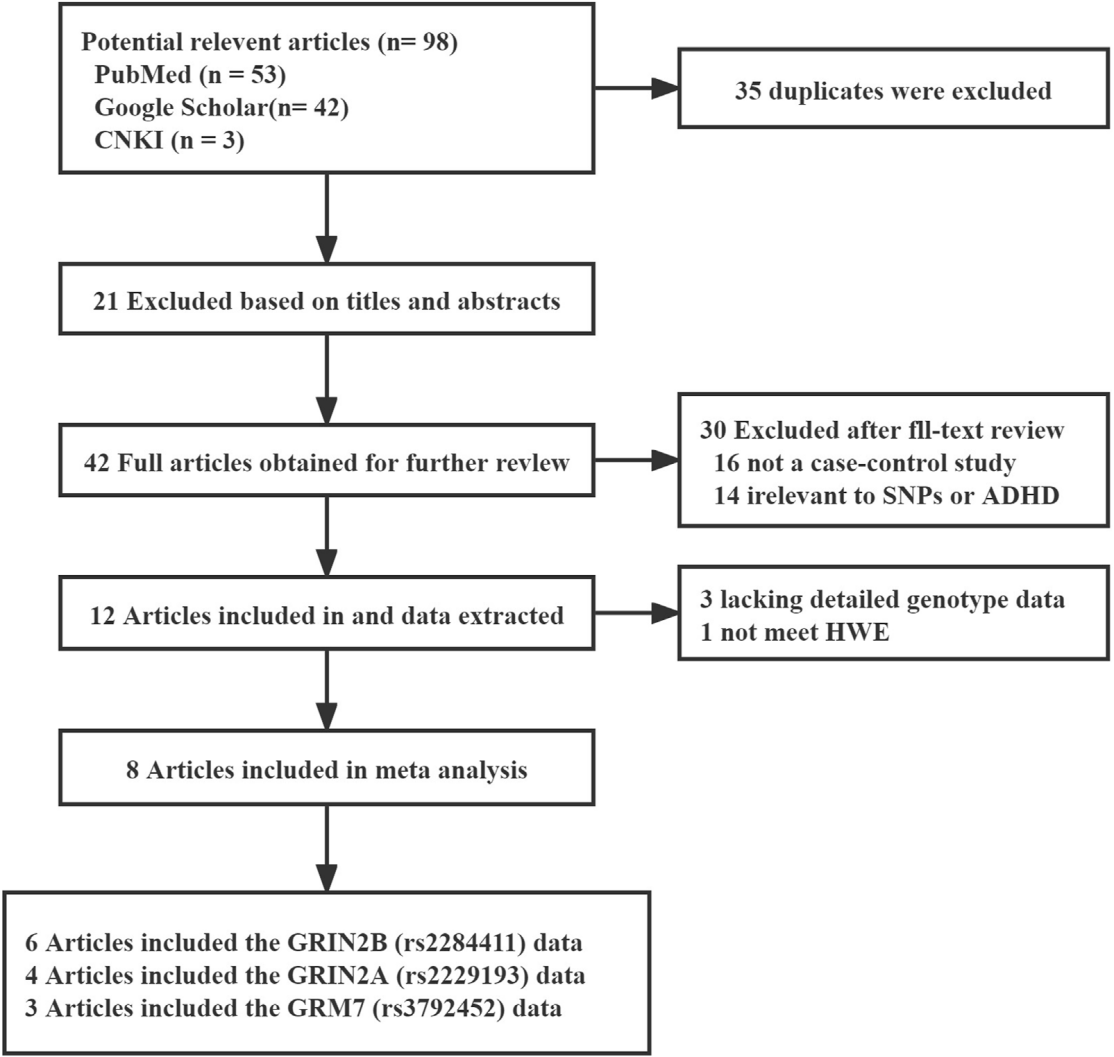
Flow chart of study selection in the meta-analysis.

A total of 98 possible studies were identified and evaluated based on the established inclusion and exclusion criteria. The study selection process is depicted in Figure 1, which illustrates the flow of the selection procedure. Six articles had data for rs2284411. Three articles had data for rs3792452. Four articles had data for rs2229193. The characteristics of each included study are shown in Table 1.

**TABLE 1:** Characteristics of each study included in the meta-analysis.

SNP					Allele distribution				Genotype distribution					
					ADHD, n		HC, n		ADHD, n			HC, n		
rs2284411	Author	Year	Origin	ADHD/HC,n	C	T	C	T	CC	CT	TT	CC	CT	TT
	Ding et al.	2021	Chinese	49/50	85	13	84	16	37	11	1	35	14	1
	Zhang et al.	2020	Chinese	396/434	664	128	708	160	278	108	10	294	120	20
	Yoo et al.	2020	Korean	47/47	78	16	71	23	33	12	2	27	17	3
	Kim et al.	2020	Korean	253/98	411	95	152	44	167	77	9	58	36	4
	Kim et al.	2018	Korean	67/44	116	18	69	19	51	14	2	26	17	1
	Park et al.	2013	Korean	201/159	322	80	265	53	128	66	7	111	43	5
rs2229193	Author	Year	Origin	ADHD/HC,n	C	T	C	T	CC	CT	TT	CC	CT	TT
	Ding et al.	2021	Chinese	49/50	90	8	89	11	41	8	0	39	11	0
	Kim et al.	2020	Korean	253/98	473	33	183	13	220	33	0	85	13	0
	Hang et al.	2019	Chinese	311/345	576	46	633	57	270	36	5	293	47	5
	Park et al.	2013	Korean	201/159	379	23	296	22	179	21	1	137	22	0
rs3792452	Author	Year	Origin	ADHD/HC,n	G	A	G	A	GG	GA	AA	GG	GA	AA
	Yoo et al.	2020	Korean	47/47	87	7	83	11	40	7	0	36	11	0
	Carmen et al.	2017	Spanish	286/338	432	140	523	153	170	92	24	204	115	19
	Park et al.	2013	Korean	202/159	373	29	299	19	174	25	2	140	19	0

n–Number; ADHD–Attention-deficit/hyperactivity disorder; HC–healthy controls.

### 3.2 Heterogeneity analysis

#### 3.2.1 rs2284411

Overall, moderate heterogeneity among studies (Park et al., 2013; Kim et al., 2018; Kim et al., 2020; Yoo et al., 2020; DING et al., 2021; Zhang et al., 2021) was detected in the dominant (TT + CT vs. CC: I^2^ = 30.8%; *p* = 0.205), overdominant (CT vs. TT + CC: I^2^ = 33.8%; *p* = 0.182), and heterozygous (CT vs. CC: I^2^ = 34.8%; *p* = 0.176) models; low heterogeneity was detected in the allele model (T vs. C: I^2^ = 13.0%; *p* = 0.331); no heterogeneity was detected in the recessive (TT vs. CC + CT: I^2^ = 0.0%; *p* = 0.914) and homozygous (TT vs. CC: I^2^ = 0.0%; *p* = 0.904) models (Table 2).

**TABLE 2:** Heterogeneity analysis with fixed-effect model.

SNP	Group	Genetic model	N	I^2^	*P*
rs2284411	overall	TT + CT vs. CC	6	30.80%	0.205
		TT vs. CC + CT	6	0.00%	0.914
		CT vs. TT + CC	6	33.80%	0.182
		TT vs. CC	6	0.00%	0.904
		CT vs. CC	6	34.80%	0.176
		T vs C	6	13.00%	0.331
	Chinese subgroup	TT + CT vs. CC	2	0.00%	0.732
		TT vs. CC + CT	2	0.00%	0.664
		CT vs. TT + CC	2	0.00%	0.573
		TT vs. CC	2	0.00%	0.696
		CT vs. CC	2	0.00%	0.616
		T vs. C	2	0.00%	0.887
	Korean subgroup	TT + CT vs. CC	4	57.70%	0.069
		TT vs. CC + CT	4	0.00%	0.955
		CT vs. TT + CC	4	57.20%	0.072
		TT vs. CC	4	0.00%	0.899
		CT vs. CC	4	58.80%	0.064
		T vs. C	4	46.90%	0.13
rs2229193	overall	TT + CT vs. CC	4	0.00%	0.935
		TT vs. CC + CT	4	0.00%	0.663
		CT vs. TT + CC	4	0.00%	0.92
		TT vs. CC	4	0.00%	0.669
		CT vs. CC	4	0.00%	0.919
		T vs. C	4	0.00%	0.954
rs3792452	overall	AA+ GA vs. GG	3	0.00%	0.522
		AA vs. GG + GA	3	0.00%	0.546
		GA vs. AA+ GG	3	0.00%	0.626
		AA vs. GG	3	0.00%	0.537
		GA vs. GG	3	0.00%	0.605
		A vs. G	3	0.00%	0.475

I ^ 2: measure to quantify the degree of heterogeneity in meta-analyses.

In the subgroup, there was no heterogeneity among studies (DING et al., 2021; Zhang et al., 2021) in the dominant (TT + CT vs. CC: I^2^ = 0.0%; *p* = 0.732), recessive (TT vs. CC + CT: I^2^ = 0.0%; *p* = 0.664), overdominant (CT vs. TT + CC: I^2^ = 0.0%; *p* = 0.573), homozygous (TT vs. CC: I^2^ = 0.0%; *p* = 0.696), heterozygous (CT vs. CC: I^2^ = 0.0%; *p* = 0.616) and allele (T vs. C: I^2^ = 0.0%; *p* = 0.887) models in the Chinese population. In the Korean population, moderate heterogeneity was detected among studies (Park et al., 2013; Kim et al., 2018; Kim et al., 2020; Yoo et al., 2020) in the dominant (TT + CT vs. CC: I^2^ = 57.7%; *p* = 0.069), overdominant (CT vs. TT + CC: I^2^ = 57.2%; *p* = 0.072), heterozygous (CT vs. CC: I^2^ = 58.8%; *p* = 0.064) and allele (T vs. C: I^2^ = 46.9%; *p* = 0.130) models, and no heterogeneity was detected in the recessive (TT vs. CC + CT: I^2^ = 0.0%; *p* = 0.955) and homozygous (TT vs. CC: I^2^ = 0.0%; *p* = 0.899) models (Table 2).

#### 3.2.2 rs2229193

No heterogeneity among studies (Park et al., 2013; Huang, 2019; Kim et al., 2020; DING et al., 2021) was detected in the dominant (TT + CT vs. CC: I^2^ = 0.0%; *p* = 0.935), recessive (TT vs. CC + CT: I^2^ = 0.0%; *p* = 0.663), overdominant (CT vs. TT + CC: I^2^ = 0.0%; *p* = 0.920), homozygous (TT vs. CC: I^2^ = 0.0%; *p* = 0.669), heterozygous (CT vs. CC: I^2^ = 0.0%; *p* = 0.919) and allele (T vs. C: I^2^ = 0.0%; *p* = 0.954) models (Table 2).

#### 3.2.3 rs3792452

No heterogeneity among studies (Park et al., 2013; Carmen, 2017; Yoo et al., 2020) was detected in the dominant (AA+ GA vs. GG: I^2^ = 0.0%; *p* = 0.522), recessive (AA vs. GG + GA: I^2^ = 0.0%; *p* = 0.546), overdominant (GA vs. AA+ GG: I^2^ = 0.0%; *p* = 0.626), homozygous (AA vs. GG: I^2^ = 0.0%; *p* = 0.537), heterozygous (GA vs. GG: I^2^ = 0.0%; *p* = 0.605) and allele (A vs. G: I^2^ = 0.0%; *p* = 0.475) models (Table 2).

### 3.3 Sensitivity analysis

**FIGURE 2:**
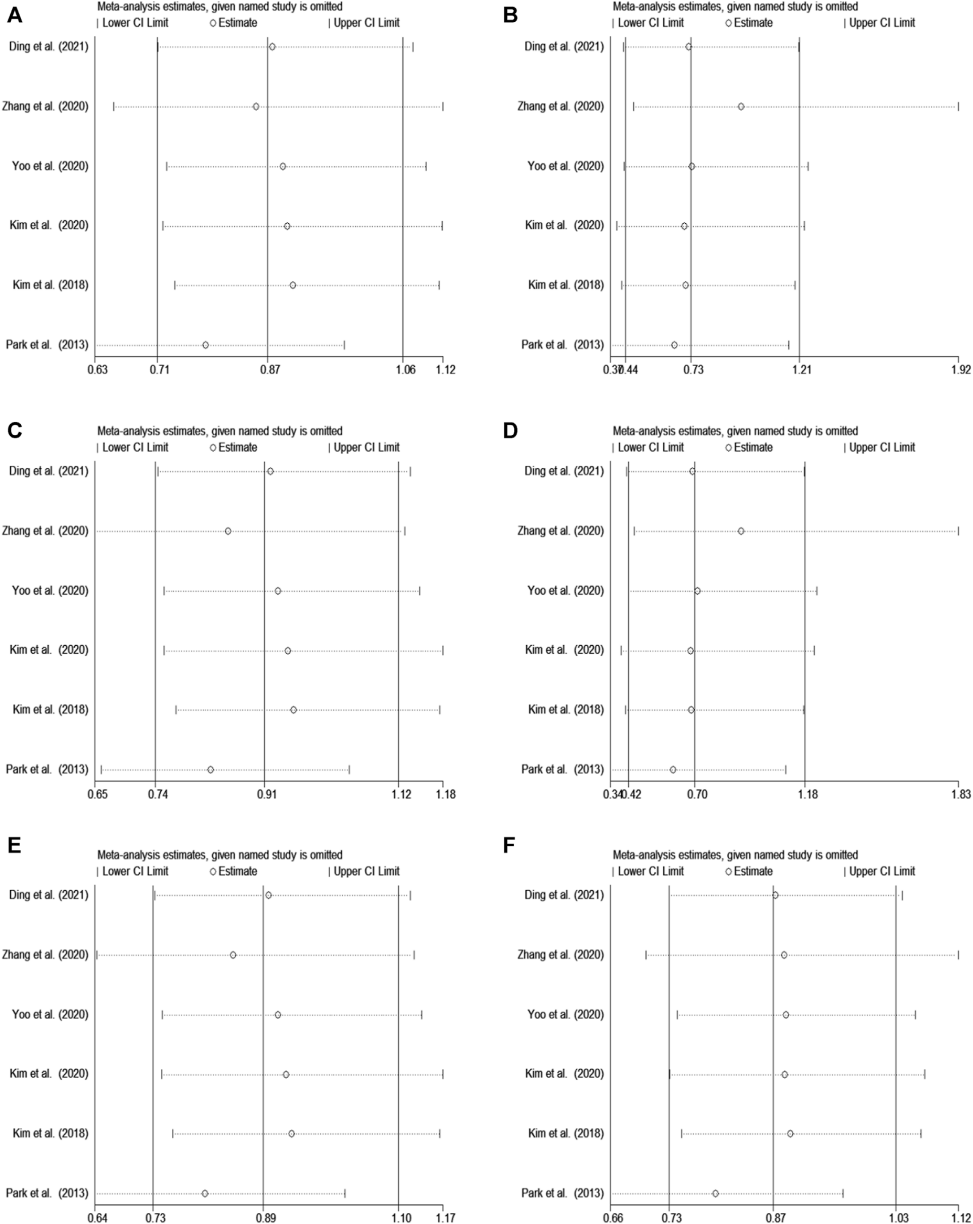
Sensitivity analysis was performed by removing one study at a time in rs2284411. **(A)** Dominant model, TT + CT vs. CC. **(B)** Recessive model, TT vs. CC + CT. **(C)** Overdominant model, CT vs. TT + CC. **(D)** Homozygous model, TT vs. CC. **(E)** Heterozygous model, CT vs. CC. **(F)** Allele model, T vs. C

**FIGURE 3:**
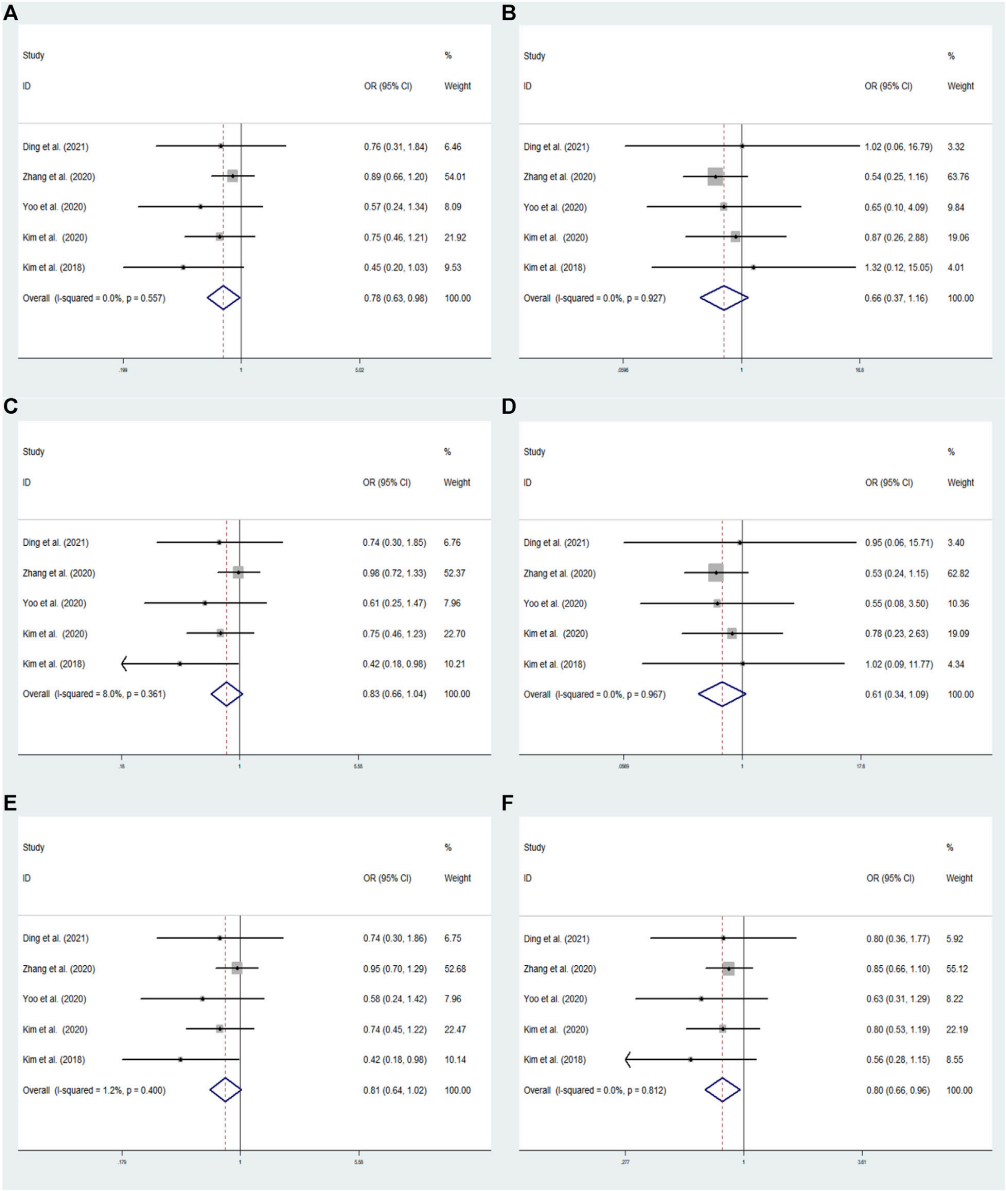
The meta-analysis for the association between GRIN2B rs2284411 and ADHD susceptibility with a fixed effects model in overall. **(A)** Dominant model: TT + CT vs. CC. **(B)** Recessive model: TT vs. CC + CT. **(C)** Overdominant model: CT vs. TT + CC. **(D)** Homozygous model: TT vs. CC. **(E)** Heterozygous model: CT vs. CC. **(F)** Allele model: T vs. C. OR: odds ratio, CI: confidence interval, I^2^: measurement to quantify the degree of heterogeneity in meta-analyses.

In rs2284411, we conducted a sensitivity analysis with a fixed effectsmodel to assess the impact of each study on the overall OR of the six genetic models and to investigate the sources of moderate heterogeneity. The results are depicted in Figure 2. The exclusion of Park’s study significantly decreased heterogeneity, notably in the dominant (TT + CT vs. CC: I^2^ = 0.0%; *p* = 0.557) and allele (T vs. C: I^2^ = 0.0%; *p* = 0.812) models; Low heterogeneity was detected in the in the overdominant (CT vs. TT + CC: I^2^ = 8%; *p* = 0.361) and heterozygous (CT vs. CC: I^2^ = 1.2%; *p* = 0.400) models (Figure 3). Therefore, for the stability of results, the meta-analysis of GRIN2B (rs2284411) and ADHD did not include Park’s study.

### 3.4 Overall meta-analysis results

#### 3.4.1 rs2284411

In overall analysis, using a fixed effects model, the GRIN2B rs2284411 was shown to be significantly associated with decreased ADHD risk for the dominant (TT + CT vs. CC: OR = 0.783; 95% CI: 0.627–0.980; *p* = 0.032) and allele (T vs. C: OR = 0.795; 95% CI: 0.656–0.964; *p* = 0.019) models; No significant associations were identified under the overdominant (CT vs. TT + CC: OR = 0.826; 95% CI: 0.656–1.040; *p* = 0.104), heterozygous (CT vs. CC: OR = 0.807; 95% CI: 0.640–1.018; *p* = 0.070), homozygous (TT vs. CC: OR = 0.614; 95% CI: 0.345–1.094; *p* = 0.098), and recessive (TT vs. CC + CT: OR = 0.658; 95% CI: 0.372–1.165; *p* = 0.151) models (Figure 3).

#### 3.4.2 rs2229193

**FIGURE 4:**
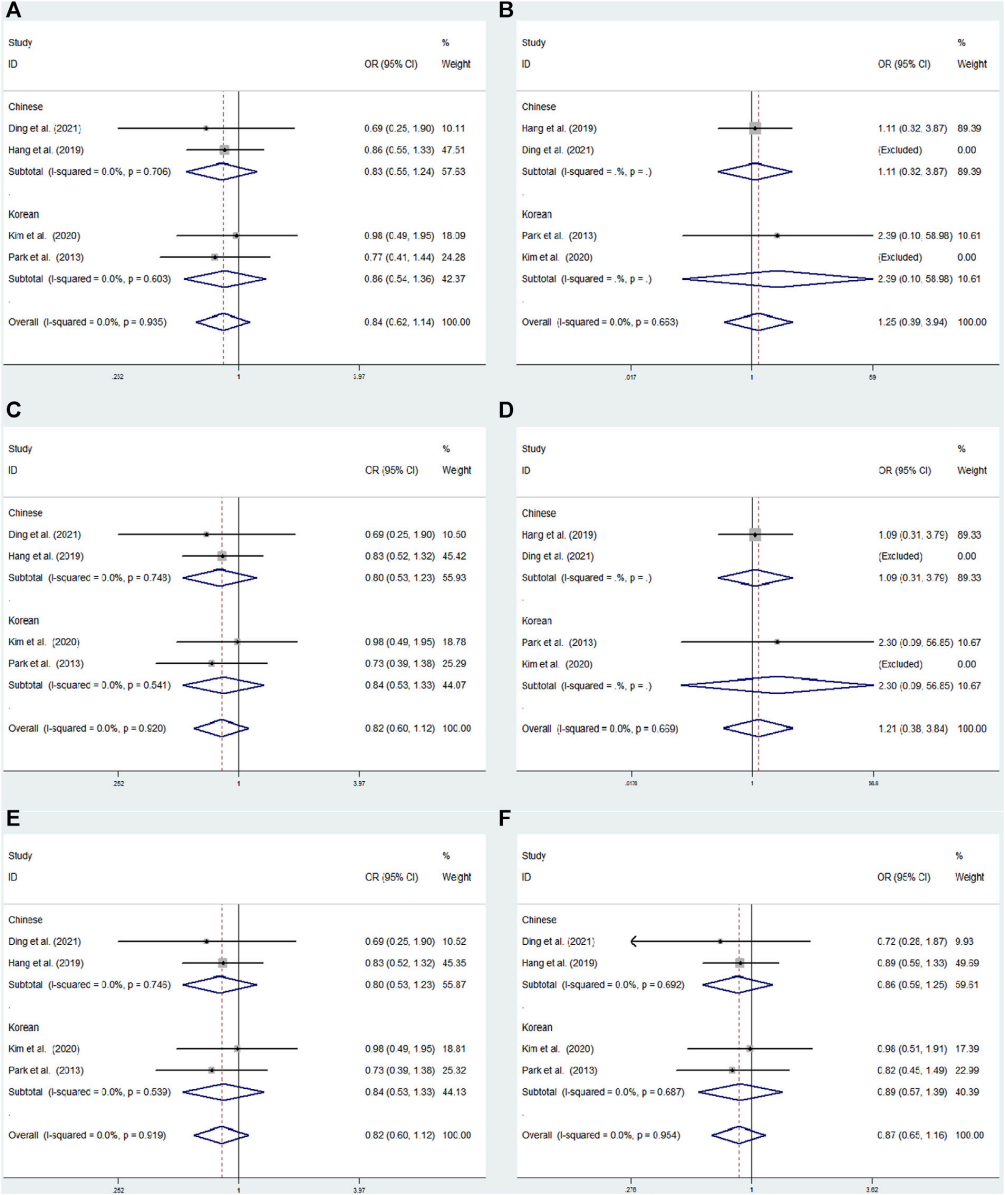
The meta-analysis for the association between GRIN2A rs2229193 and ADHD susceptibility with a fixed effects model. **(A)** Dominant model: TT + CT vs. CC. **(B)** Recessive model: TT vs. CC + CT. **(C)** Overdominant model: CT vs. TT + CC. **(D)** Homozygous model: TT vs. CC. **(E)** Heterozygous model: CT vs. CC. **(F)** Allele model: T vs. C. OR: odds ratio, CI: confidence interval, I^2^: measurement to quantify the degree of heterogeneity in meta-analyses.

In overall analysis, no significant associations were observed under the dominant (TT + CT vs. CC: OR = 0.840; 95% CI: 0.619–1.139;*p* = 0.261), overdominant (CT vs. TT + CC: OR = 0.819; 95% CI: 0.599–1.119; *p* = 0.210), recessive (TT vs. CC + CT: OR = 1.246; 95% CI: 0.395–3.937; *p* = 0.707), heterozygous (CT vs. CC: OR = 0.819; 95% CI: 0.599–1.120; *p* = 0.211), allele (T vs. C: OR = 0.871; 95% CI: 0.654–1.158; *p* = 0.341), and homozygous (TT vs. CC: OR = 1.215; 95% CI: 0.384–3.842; *p* = 0.741) models (Figure 4).

#### 3.4.3 rs3792452

**FIGURE 5:**
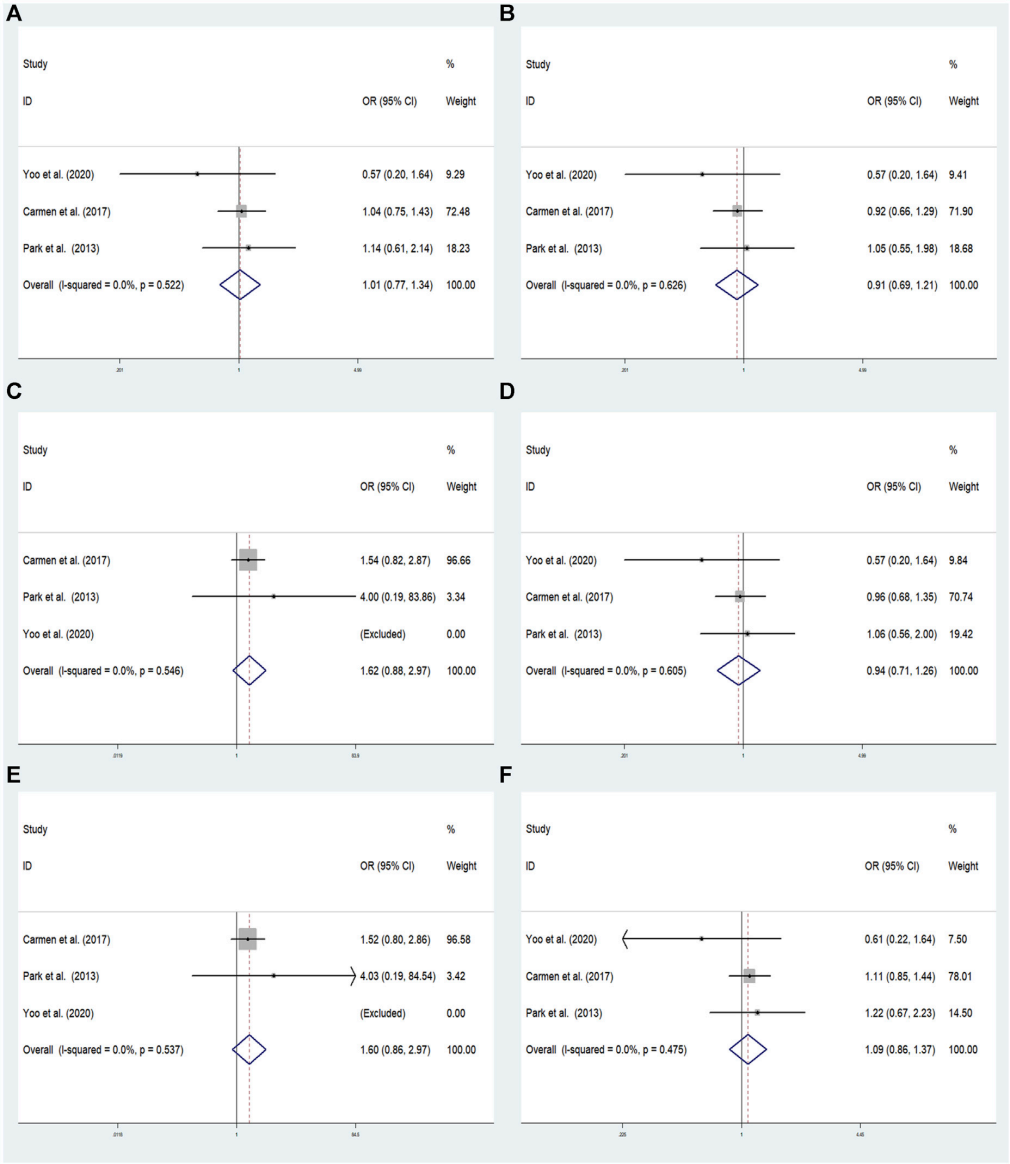
The meta-analysis for the association between GRM7 rs3792452 and ADHD susceptibility with a fixed effects model. **(A)** Dominant model: AA+ GA vs. GG. **(B)** Recessive model: AA vs. GG + GA. **(C)** Overdominant: GA vs. AA+ GG. **(D)** Homozygous: AA vs. GG. **(E)** Heterozygous model: GA vs. GG. **(F)** Allele model: A vs. G. OR: odds ratio, CI: confidence interval, I^2^: measurement to quantify the degree of heterogeneity in meta-analyses.

In overall analysis, there were no significant associations found under the dominant (AA+ GA vs. GG: OR = 1.015; 95% CI: 0.771–1.336; *p* = 0.918), overdominant (GA vs. AA+ GG: OR = 0.911; 95% CI: 0.685–1.210; *p* = 0.519), recessive (AA vs. GG + GA:OR = 1.620; 95% CI: 0.883–2.973; *p* = 0.119), heterozygous (GA vs. GG: OR = 0.941; 95% CI: 0.705–1.256; *p* = 0.680), homozygous (AA vs. GG: OR = 1.602; 95% CI: 0.864–2.971; *p* = 0.135), and allele (A vs. G: OR = 1.087; 95% CI: 0.861–1.372; *p* = 0.483) models (Figure 5).

### 3.5 Subgroup analyses

#### 3.5.1 rs2284411

**FIGURE 6:**
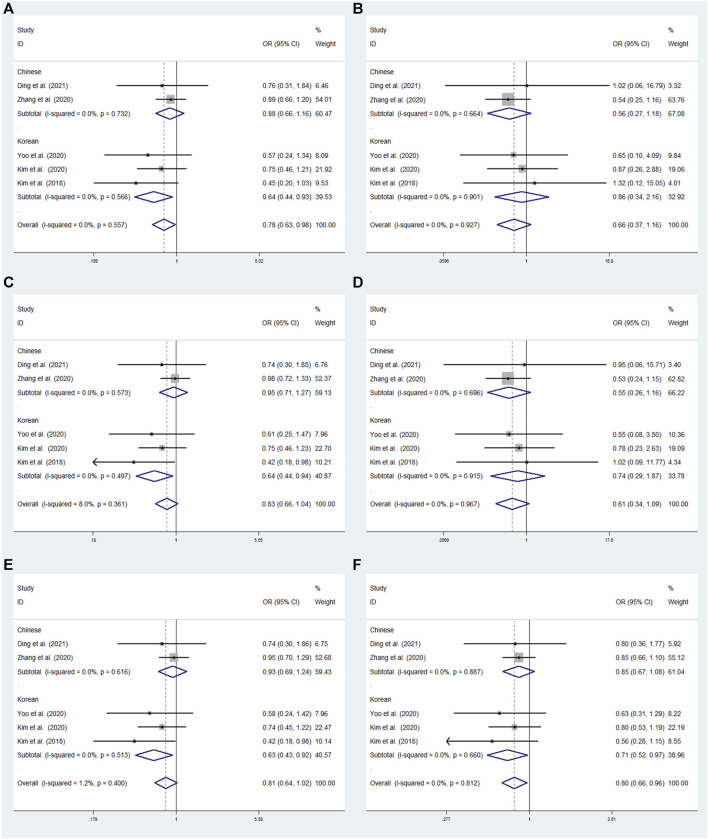
Subgroup meta-analysis for the association between GRIN2B rs2284411 and ADHD susceptibility with a fixed effects model. **(A)** Dominant model: TT + CT vs. CC. **(B)** Recessive model: TT vs. CC + CT. **(C)** Overdominant model: CT vs. TT + CC. **(D)** Homozygous model: TT vs. CC. **(E)** Heterozygous model: CT vs. CC. **(F)** Allele model: T vs. C. OR: odds ratio, CI: confidence interval, I^2^: measurement to quantify the degree of heterogeneity in meta-analyses.

Subgroup analysis was conducted by origin to evaluate the association between rs2284411 and ADHD susceptibility in six models. In the Korean subgroup, rs2284411 was significantly associated with a decreased risk of ADHD for the dominant (TT + CT vs. CC: OR = 0.640; 95% CI: 0.442–0.928; *p* = 0.019), overdominant (CT vs. TT + CC: OR = 0.641; 95% CI: 0.438–0.938; *p* = 0.022), heterozygous (CT vs. CC: OR = 0.630; 95% CI: 0.429–0.925; *p* = 0.018) and allele (T vs. C: OR = 0.712; 95% CI: 0.521–0.974; *p* = 0.034) models; No significant associations were identified under the recessive (TT vs. CC + CT: OR = 0.858; 95% CI: 0.342–2.155; *p* = 0.745) and homozygous (TT vs. CC: OR = 0.740; 95% CI: 0.292–1.875; *p* = 0.525) models (Figure 6). In the Chinese subgroup, No significant associations were detected in rs2284411 under the recessive (TT vs. CC + CT: OR = 0.560; 95% CI: 0.267–1.176; *p* = 0.126), dominant (TT + CT vs. CC: OR = 0.877; 95% CI: 0.663–1.160; *p* = 0.358), homozygous (TT vs. CC: OR = 0.550; 95% CI: 0.261–1.161; *p* = 0.117), overdominant (CT vs. TT + CC: OR = 0.954; 95% CI: 0.714–1.274; *p* = 0.751), allele (T vs. C: OR = 0.848; 95% CI: 0.665–1.082; *p* = 0.184), and heterozygous (CT vs. CC: OR = 0.928; 95% CI: 0.694–1.242; *p* = 0.616) models using a fixed effects model (Figure 6).

#### 3.5.2 rs2229193

We conducted a subgroup analysis by origin to assess the correlation between SNPs and susceptibility to ADHD in the six models. No significant associations have been found in rs2229193 under the dominant (TT + CT vs. CC: OR = 0.827; 95% CI: 0.552–1.239; *p* = 0.357), overdominant (CT vs. TT + CC: OR = 0.804; 95% CI: 0.527–1.226; *p* = 0.311), heterozygous (CT vs. CC: OR = 0.805; 95% CI: 0.528–1.228; *p* = 0.314) and allele (T vs. C: OR = 0.859; 95% CI: 0.592–1.247; *p* = 0.424) models using a fixed effects model in the Chinese subgroup (Figure 5). In the Korean subgroup, no significant relationships under the dominant (TT + CT vs. CC: OR = 0.875; 95% CI: 0.540–1.362; *p* = 0.515), overdominant (CT vs. TT + CC: OR = 0.838; 95% CI: 0.526–1.334; *p* = 0.456), heterozygous (CT vs. CC: OR = 0.837; 95% CI: 0.562–1.333; *p* = 0.454) and allele (T vs. C: OR = 0.888; 95% CI: 0.569–1.385; *p* = 0.600) model using a fixed effects model (Figure 4).

### 3.6 Publication bias

**FIGURE 7:**
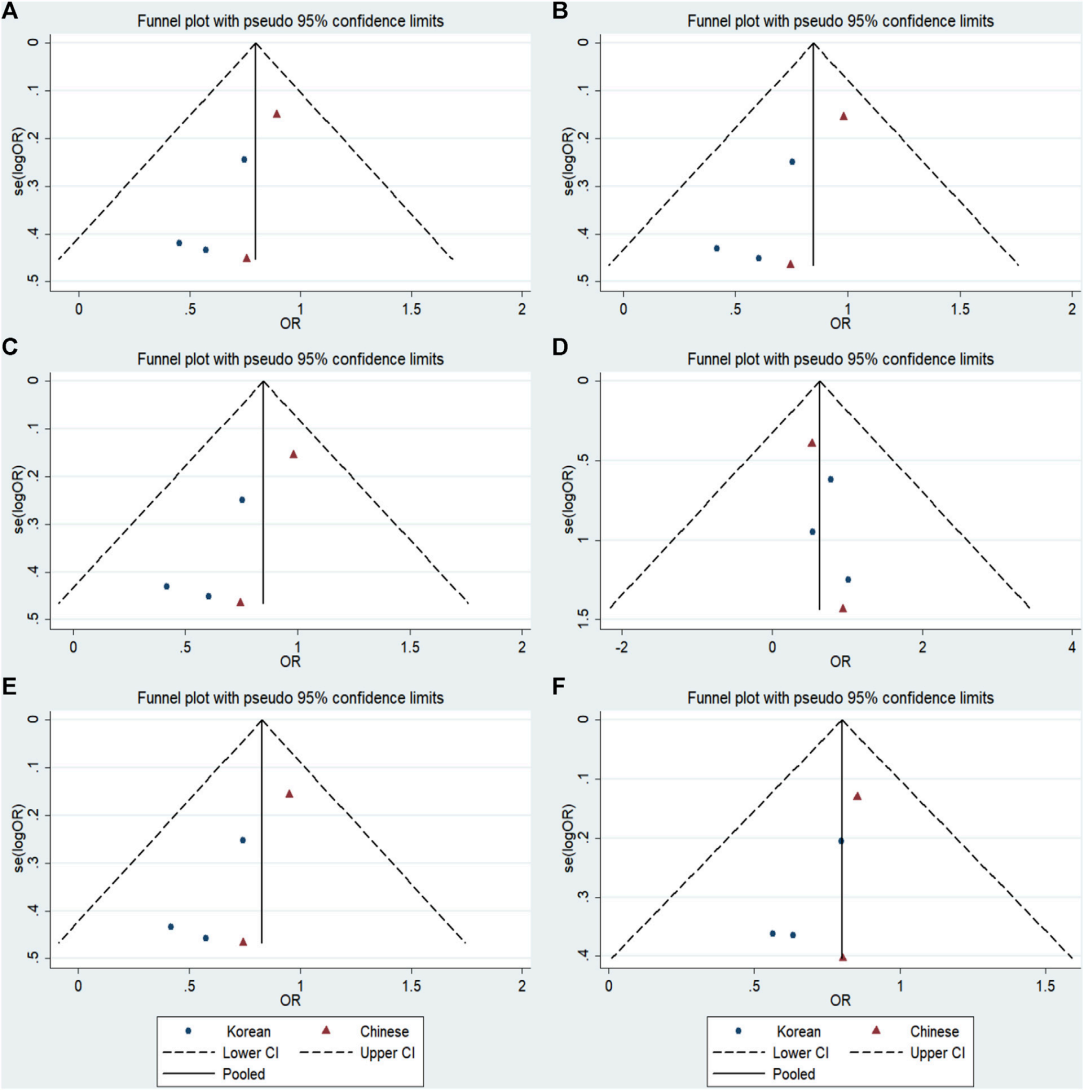
Funnel plot of the odds ratios in the GRIN2B rs2284411 meta-analysis. **(A)** Dominant model: TT + CT vs. CC. **(B)** Recessive model: TT vs. CC + CT. **(C)** Overdominant model: CT vs. TT + CC. **(D)** Homozygous model: TT vs. CC. **(E)** Heterozygous model: CT vs. CC. **(F)** Allele model: T vs. C.

**FIGURE 8:**
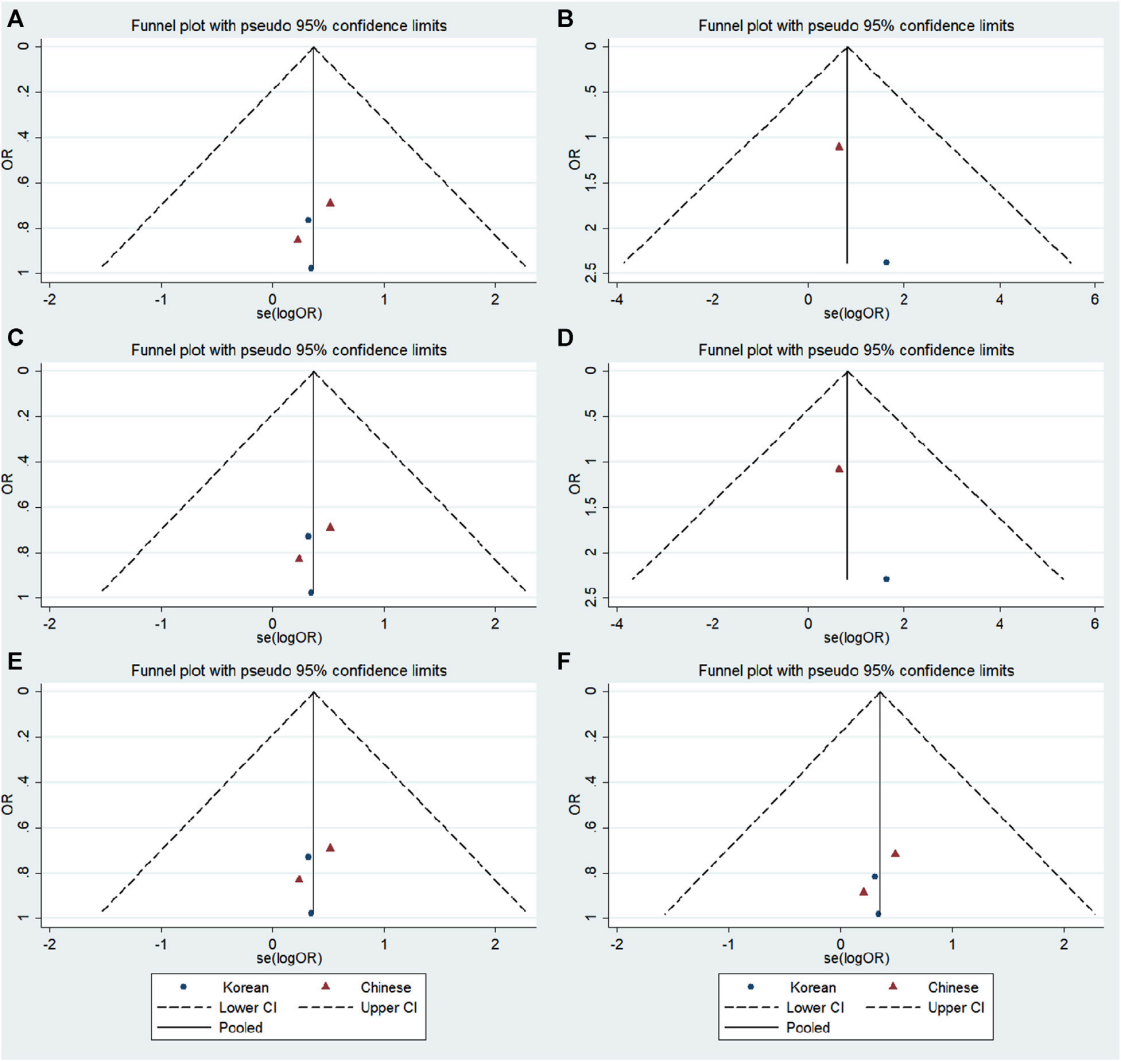
Funnel plot of the odds ratios in the GRIN2A rs2229193 meta-analysis. **(A)** Dominant model: TT + CT vs. CC. **(B)** Recessive model: TT vs. CC + CT. **(C)** Overdominant model: CT vs. TT + CC. **(D)** Homozygous model: TT vs. CC. **(E)** Heterozygous model: CT vs. CC. **(F)** Allele model: T vs. C.

**FIGURE 9:**
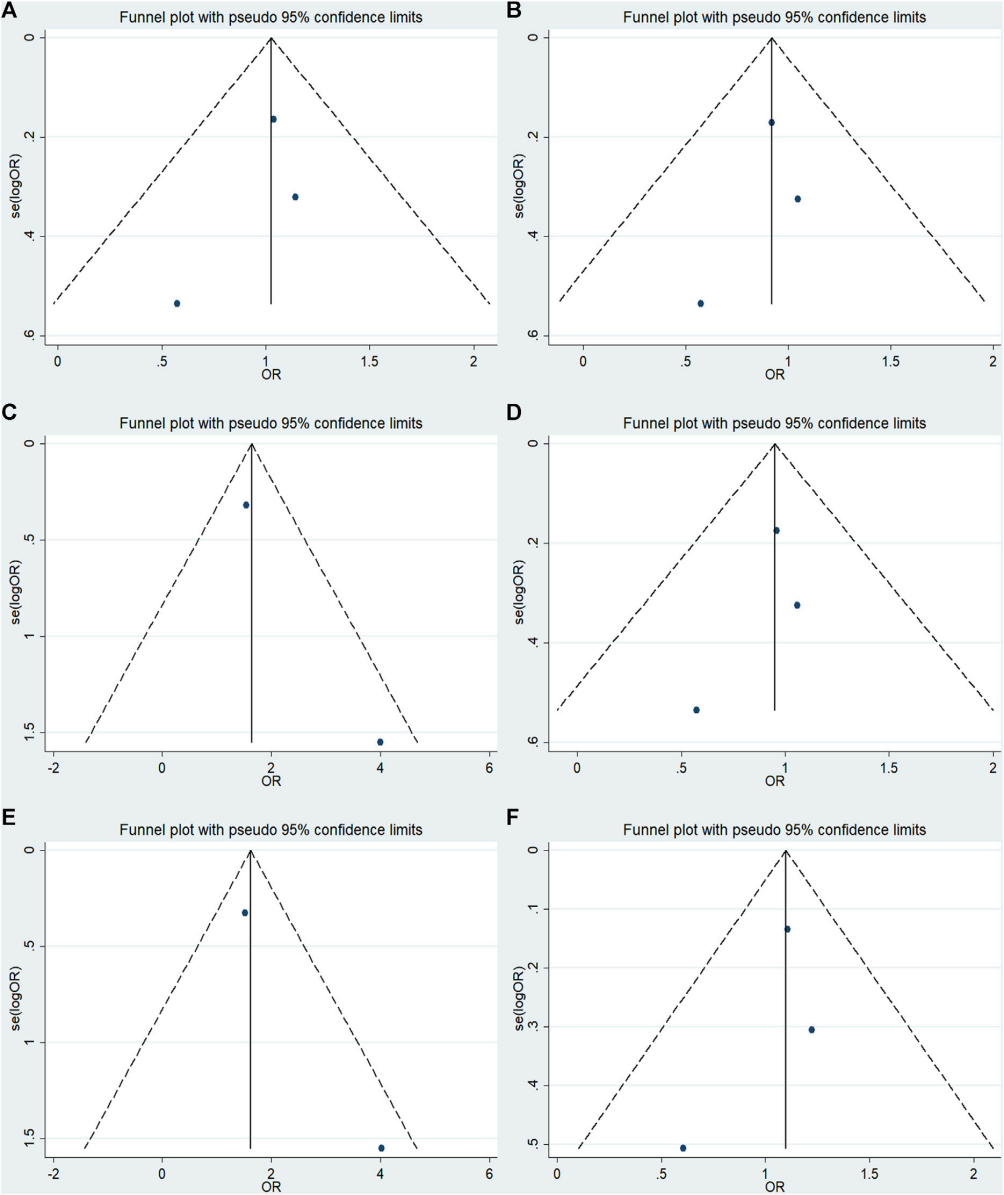
Funnel plot of the odds ratios in the GRM7 rs3792452 meta-analysis.**(A)** Dominant model: AA+ GA vs. GG. **(B)** Recessive model: AA vs. GG + GA. **(C)** Overdominant: GA vs AA+ GG. **(D)** Homozygous: AA vs. GG. **(E)** Heterozygous model: GA vs GG. **(F)** Allele model: A vs. G.

No significant publication bias was detected in any of the genetic models based on the results from Begg’s and Egger’s tests (all *p* > 0.05, data not shown). Additionally, Figures 7–9 depict the funnel plots.

## 4 Discussion

Candidate gene studies found a role for GRIN2B rs2284411 in ADHD susceptibility, the C allele was correlated with augmented inattentive symptoms as determined by parent and teacher interviews in ADHD patients, as compared to the T allele (Dorval et al., 2007). Kim et al. reported that rs2284411 CC genotype were associated with an increased risk of attention deficit disorder in patients with ADHD (Kim et al., 2020). Interestingly, It was observed that there was a substantial correlation between rs2284411 and IQ in the control group, with the T allele exhibiting a protective effect when compared to the C allele (Kim et al., 2020). Therefore, previous studies have shown that the rs2284411 C allele of the GRIN2B gene is linked to lower IQ, more omission errors, and higher RTV (Kim et al., 2020), these results were similar to Dorval’s study showing the C allele as a risk factor for inattention (Dorval et al., 2007). Our meta-analysis has added to the evidence that rs2284411 TT + CT and T allele are linked to a decreased risk of ADHD, particularly for Koreans, meaning that the T allele might be a protective factor against the ADHD. The fact that the Korean population has a highly homogeneous genetic background originating from a single race may be related to this (Wacholder et al., 2002). The precise mechanism by which the GRIN2B variant rs2284411 is associated with ADHD has yet to be determined, as it is situated in intron 3 of the GRIN2B gene. It does not change the amino acid sequence. This variant may be in linkage disequilibrium with a functional variant or alter regulatory features of GRIN2B expression such as transcription, mRNA processing, necrotic export or secondary structure transformation (Dorval et al., 2007).

In addition, GRIN2A rs2229193 G/G homozygotes was found to be at a higher risk of attention disorders in ADHD patients, and more omission errors associated with G allele (Kim et al., 2020), but other studies have identified the A allele as the risk factor (Barnby et al., 2005; Tarabeux et al., 2011). Analysis of transmission disequilibrium tests revealed GRM7 rs3792452 G allele preferentially transmitted (Park et al., 2013), a variant that remains associated with attention deficits in ADHD (Park et al., 2013). Among the GWAS of 187 children with ADHD responding to MPH, The association with rs3792452 was the most interesting of the suggestive findings (Mick et al., 2008). Children with the rs37952452 GA genotype showed more improvement than GG after 8 weeks of MPH (Park et al., 2014). However, no significant association between rs2229193 or rs3792452 and ADHD susceptibility was found in our meta-analysis. Differences in statistical analysis methods, ethnic differences, and limited sample size, may contribute to the above. Furthermore, ADHD is the result of a combination of environmental and genetic risk factors (Faraone et al., 2015). Thus, genes may be involved in ADHD by interacting with other genes or by interacting with environmental factors. Moreover, the results could be attributed to other unidentified polymorphisms that impact gene expression and susceptibility to ADHD, or the possibility that rs2229193 and rs3792452 are in linkage disequilibrium with other SNPs linked to ADHD. Additionally, it is possible that other genes also play a role in this association (Park et al., 2013).

In addition, because genes associated with ADHD may have a smaller impact (Smalley, 1997), the GRIN2A (rs2229193) and GRM7 (rs3792452) polymorphisms are considered uncommon disease loci with smaller effects. Furthermore, the genetic models used in different studies vary widely, with some studies only using one or two models, which can lead to incomplete conclusions. In our study, we gathered all relevant research in the field and evaluated the risk as well as combined results using all genetic models. This helped us overcome the issue of conflicting results often seen in individual studies with small sample sizes. Indeed, our study found no association between the rs2229193 and rs3792452 polymorphisms and susceptibility to ADHD.

The present meta-analysis had several limitations. Firstly, there were a limited number of studies available on the rs2284411, rs2229193, and rs3792452 polymorphisms and their association with ADHD risk. Some articles did not have available data for the meta-analysis, which may have impacted the overall estimates. Specifically, there were few studies that examined the relationship between rs2229193 or rs3792452 and ADHD risk. Only four articles provided data for rs2229193, and one article did not include data for HWE. Additionally, the majority of the studies included in the analysis focused on Asian populations, with only one study on a Spanish population. Therefore, there is a need for larger sample sizes in other ethnicities around the world. Lastly, this study did not account for the effects of other risk factors and only assessed the association between rs2284411, rs2229193, or rs3792452 and ADHD risk. It is important for further research to explore the susceptibility factors for ADHD.

## 5 Conclusion

As far as we know, the role of rs2284411, rs2229193, or rs3792452 polymorphisms in ADHD was investigated in this study for the first time in a meta-analysis. The results of the meta-analysis provide sufficient evidence to suggest that rs2284411 T allele have a significant association with decreased ADHD risk, especially for Koreans.
